# Hepatic transcriptome profiling according to growth rate reveals acclimation in metabolic regulatory mechanisms to cyclic heat stress in broiler chickens

**DOI:** 10.1016/j.psj.2022.102167

**Published:** 2022-09-20

**Authors:** C. Lim, B. Lim, D.Y. Kil, J.M. Kim

**Affiliations:** Department of Animal Science and Technology, Chung-Ang University, Anseong-si, Gyeonggi-do 17546, Republic of Korea

**Keywords:** hepatic transcriptome, growth performance, acclimation, heat stress, broiler chicken

## Abstract

Climate change has numerous effects on poultry that result in welfare concerns and economic losses in agricultural industries. However, the mechanisms underlying the acclimation to heat stress in poultry have not been comprehensively defined. Therefore, identifying associated patterns of gene regulation and understanding the molecular mechanisms of acclimation to a warmer environment will provide insights into the acclimation system of broiler chickens. We profiled differentially expressed genes (**DEGs**) associated with differences in growth performance under heat stress conditions in the liver tissues of broilers based on RNA sequencing data. The DEGs were identified by comparison to the gene expression levels of broilers exhibiting average growth at 28 d of age (**D28A**) and D36A relative to those at D21A. In D36A, 507 and 312 DEGs were up- and downregulated, respectively, whereas 400 and 156 DEGs were up- and downregulated in D28A, respectively. Pathway enrichment analysis further revealed that “fatty acid degradation” and “heat shock protein expression” were upregulated in broilers exhibiting a higher growth and weight, whereas “cell cycle arrest” and “amino acid metabolism” were downregulated. Transcriptome profiling revealed that the acclimatized group supplied fat and energy from the liver to tissues through the breakdown of fatty acids. Furthermore, homeostasis was maintained via heat shock proteins and antioxidant enzymes. The characterized candidate genes and mechanisms associated with the response to heat stress might serve as a foundation for improving the ability of broilers to acclimatize under heat stress conditions.

## INTRODUCTION

Climate change negatively affects poultry, which results in considerable concerns regarding the welfare of the animals and economic losses (Yalcin et al., 2001). Rising temperatures due to global warming cause a negative balance between the net energy flowing from an animal's body to its ambient environment and the amount of heat energy generated by the animal (Cahaner and Leenstar, 1992; Cheng et al., 1997). The conditions that cause these negative influences are generally referred to as heat stress (**HS**) conditions.

Most domestic animals are warm-blooded, producing high levels of metabolic heat. Poultry are considered to be particularly vulnerable to HS because of their limited capacity to regulate the heat released by evaporation (Star et al., 2008; Mutaf et al., 2009). HS is especially burdensome for fast-growing, meat-type chickens, reducing performance, increasing mortality, and impairing reproduction (St-Pierre et al., 2003; Mashaly et al., 2004), with the concomitant decrease in meat productivity being of considerable concern. Selection for muscle development has resulted in broiler chicken with higher performance with respect to economic traits such that broiler chickens exhibit difficulties when acclimatizing to stressful environmental conditions, such as high temperature and humidity (Nardone et al., 2010). Thus, a systemic understanding of the molecular processes underlying acclimation to HS in broiler chickens is imperative to address these issues.

Genetic studies on the variations in response to HS have identified specific genes related to sensitivity and tolerance (Deeb and Cahaner 2002; Mack et al., 2013). Additionally, there have been numerous studies on the function of molecular responses to HS, including chaperone changes in various animals (Schöffl et al., 1999; Horowitz et al., 2002; Coble et al., 2014). However, the mechanisms of acclimation in broiler chickens exposed to HS have not been comprehensively defined (Yang et al., 2010). Therefore, classifying the variable response under HS conditions and investigating the differentially expressed genes (**DEGs**) are required to identify the regulatory mechanisms of the acclimation system in broiler chickens.

The liver plays various crucial roles in maintaining homeostasis via energy metabolism, fat metabolism, and immunity (Geraert et al., 1996). Additionally, lipid metabolism constitutes a major function of the liver in broiler chickens because lipogenesis occurs primarily in this organ in poultry, unlike in mammals where lipogenesis occurs in the adipose tissue. Previous studies have shown that the liver is more vulnerable to oxidative stress than other organs, owing to its role in increasing the production of antioxidants to maintain homeostasis (Lin et al., 2006; Sánchez-Valle et al., 2012). The liver also regulates the levels of circulating nutrients, including those of glucose and triglycerides, which are generally linked to growth performance under HS conditions (Bauman and Currie 1980; Sakomura, 2004).

In this study, we investigated the molecular system of HS acclimation in broiler chickens based on hepatic transcriptome changes associated with exposure to cyclic HS. The DEG profiles were generated according to differences in BW (g) during broiler chicken growth to classify the genes that were modulated in response to thermal stress. Then, bioinformatic analysis was used to identify and characterize the different transcriptomic responses according to sensitivity and tolerance to HS, revealing the adaptive responses related to the acclimation mechanism in broiler chickens.

## MATERIALS AND METHODS

### Experimental Animals and Tissue Collection

**Figure 1:**
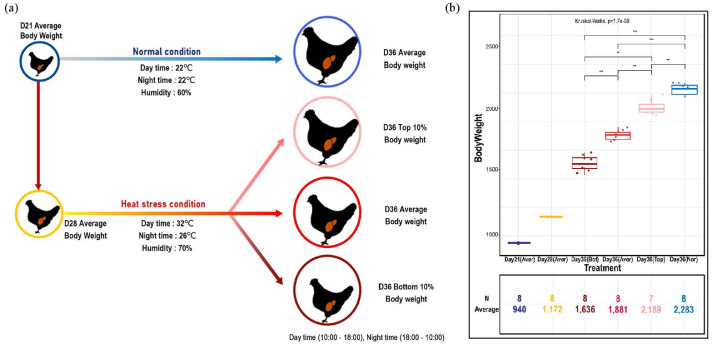
Experimental design and BW measurements. (A) Schematic experimental design showing heat stress (HS) response at difference timepoints (day 21 [D21], day 28 [D28], and day 36 [D36]) and between individuals stratified by BW (D36N, D36T [top 10%], D36A [average], and D36B [bottom 10%]). (B) Box plot showing the BW and numbers for each group. The significance was calculated using the Kruskal–Wallis test (significance levels: *P  <  0.05; **P  <  0.01; ***P  <  0.001).

The research protocol for the present experiment was approved by the Institutional Animal Care and the Use Committee (**IACUC**) at Chung-Ang University (approval number: 2019-00086). A total of 500 one-day-old Ross 308 male broiler chicks were obtained and raised according to the general guidelines of Ross 308 broiler management until 20 d of age. At 21 d of age, broilers were weighed, and 200 broilers with a close-to-average BW were finally selected. The selected chickens were allotted to 20 battery cages, which were randomly assigned to 2 different temperature groups of thermoneutral (**TN**) and HS conditions with 10 replicates per group. For TN conditions, broilers were raised at 22°C and 60% relative humidity (**RH**) from 21 d to 35 d of age. The broilers raised under HS conditions were housed at 32°C and 70% RH from 21 d to 28 d and 30°C and 70% RH from 29 d to 36 d of age from 10:00 to 18:00 h. From 18:00 to 10:00 h, the temperature was reduced to 26°C with 60% RH from 21 d to 28 d and 24°C and 60% RH from 29 d to 35 d of age. This intended change in ambient temperature was designed to simulate cyclic HS. On days 21 (**D21A**) and 28 (**D28A**), one broiler with similar BW to the total average BW was selected from each replicate (i.e., 10 birds per treatment). Based on the BW difference at 36 d of age, the broilers were divided into top 10% (**D36T**), average (**D36A**), and bottom 10% (**D36B**) groups, and one broiler was selected in each group for each replicate. For TN (**D36N**), one broiler with a similar BW to the total average BW was selected for each replicate. The selection procedure for broilers is presented in Figure 1A. Two birds with the lowest or highest BW were discarded prior to the final analysis to obtain more homogenous results. Liver tissues were collected from all groups. Forty-eight broiler chickens were selected and euthanized by CO_2_ asphyxiation. Liver samples were immediately collected, snap-frozen using liquid nitrogen, and stored at −80°C for RNA isolation.

### Library Construction and Sequencing

Total RNA was extracted from the liver tissues according to the manufacturer's recommendations using TRIzol reagent (Invitrogen, Waltham, MA). The RNA concentrations were measured using Quant-IT RiboGreen (Invitrogen). To evaluate the RNA-integrity number, samples were evaluated using the TapeStation RNA Screentape System (Agilent Technologies, Santa Clara, CA; Supplementary file 1). The cDNA library was separately prepared with 1 µg of total RNA for each sample using the Illumina TruSeq Stranded mRNA Sample Prep Kit (Illumina Inc., San Diego, CA). The first step in the workflow involved removing rRNA from the total RNA using the Ribo-Zero rRNA Removal Kit (Illumina Inc.). Subsequently, the remaining mRNA was fragmented into small pieces using divalent cations under elevated temperature conditions. The cleaved RNA fragments were copied into first-strand cDNA using SuperScript II reverse transcriptase (Invitrogen) and random primers. This step was followed by second-strand cDNA synthesis using DNA Polymerase I, RNase H, and dUTP. The cDNA fragments were subjected to an end-repair process involving the addition of a single “A” base, after which adapters were ligated. The products were then purified and enriched by PCR to create the final cDNA library. According to the qPCR Quantification Protocol Guide, the libraries were quantified using KAPA Library Quantification Kits for Illumina Sequencing platforms (Roche, Basel, Switzerland). The libraries were validated using the TapeStation D1000 ScreenTape System (Agilent Technologies). The indexed libraries were then analyzed on an Illumina HiSeq4000 instrument (Illumina Inc.), and paired-end (2  ×  150 bp) sequencing was performed.

### Data Processing and DEG Analyses

The quality of raw read data for each sample was checked using FastQC software v0.11.7 (Andrews, 2010). The reads were trimmed with adaptors using Trimmomatic v0.38 based on the quality results (Bolger et al., 2014). Subsequently, the trimmed reads were mapped to the reference genome (*GRCg6a, GCA_000002315.5*) of the Ensembl genome browser (http://www.ensembl.org/Gallus_gallus/) using HISAT2 v2.1.0 (Kim et al., 2015). Raw counts in each library were computed based on the exons in *Gallus* GTF v101 (Ensembl) using the featureCounts of the Subread package v1.6.3 (Liao et al., 2014). Whole DEG analyses for the obtained raw counts were performed using ‘edgeR v3.26.5’ in R (Robinson, et al., 2010), and the raw counts were normalized using the trimmed mean of M-value method. In the different timepoint designs, DEGs were identified by comparing the gene expression levels at D28A and D36A relative to those at D21A. On d 36, DEGs were selected by comparing the gene expression levels at D36T, D36A, and D36B relative to those at D36N, and *P*-values were corrected for multiple comparisons based on the false discovery rate. The DEGs were those with false discovery rate < 0.05 and an absolute log_2_ fold-change (**FC**) of ≥ 1. Multidimensional scaling (**MDS**) was performed using the R package ‘limma’ to demonstrate similarity between samples via gene expression patterns (Smyth, 2005).

### Functional Enrichment Analyses and DEG Clustering

The DEGs were annotated to biological processes in Gene Ontology (**GO**) terms and Kyoto Encyclopedia of Genes and Genomes (**KEGG**) pathways using the Database for Annotation, Visualization, and Integrated Discovery v6.8.GO (Dennis et al., 2003). Annotations were filtered with the DIRECT option and used for enrichment analyses with the following cut-offs: *P*-value < 0.1 and counts ≥ 2. The KEGG annotations were also enriched using the same cut-off criteria and are represented by the −log_10_ *P*-value and fold enrichment. Enriched GO terms were combined with related terms and visualized with treemaps using REVIGO (Supek et al., 2011). The most significant GO terms in the combined group are shown as representative. *Gallus* was used for all enrichment analyses. To identify the genes that were upregulated compared to those in other groups (D36A, D36B) in the D36T group on d 36, the Multiple Experiment Viewer was used (Howe et al., 2010). Modulations of the responsible genes in the selected representative KEGG pathways were confirmed using the ‘clusterProfiler’ package in R (Yu et al., 2012).

### Quantitative Real-Time PCR Validation and Protein–Protein Interaction Network Analysis

Quantitative real-time PCR (**qPCR**) was performed to confirm the expression of genes that were particularly elevated in the D36T group among the common genes in the 3 groups. Specific primers for the target genes were designed using Primer3 software (Untergasser et al., 2012). Total RNA (1 μg) was used for cDNA synthesis with the Revertaid First Strand cDNA Synthesis Kit (Thermo Fisher Scientific, Waltham, MA), according to the manufacturer's instructions. The PCR reaction was performed using a CFX connect real-time PCR Detection system (BioRad, Hercules, CA) with TOPreal qPCR 2X PreMIX (Thermo Fisher Scientific). The reaction conditions were as follows: 95°C for 10 min, followed by 45 cycles at 95°C for 10 s, 61°C for 15 s, and 72°C for 30 s, and a final step at 95°C for 10 s. The relative quantification of gene expression was calculated using the 2^−ΔΔCt^ method including normalization with the glyceraldehyde-3-phosphate dehydrogenase (***GAPDH***) gene (Livak and Schmittgen, 2001). The protein–protein interaction (**PPI**) network was constructed for the genes that were upregulated in D36T compared to the expression in other groups (D36A, D36B) using STRING v11.5 to predict PPIs.

## RESULTS

### Determination of BW Differences and Data Processing

The BW of broilers was used a selection parameter to examine the acclimation responses to HS treatments. The BW was 940 ± 5.4 g at D21A, 1,172 ± 4.5 g at D28A, and 1,881 ± 42.5 g at D36A, and the BW in the different growth performance groups on day 36 was 1,636 ± 63.9 g in D36B, 1.881 ± 42.5 g in D36A, 2,189 ± 67.0 g in D36T, and 2,283 ± 46.8 g in D36A. Evaluation using the Kruskal–Wallis test revealed significant differences among the day-36 groups (*P*-value = 1.7e^−08^; Figure 1B).

A total of 960 million paired-end sequence reads were produced from 48 liver tissues. The average number of raw reads was 22.4 million with a trimming rate of 5.8% ± 1.2. The average unique mapping rate and overall alignment rate were 86.43 and 95.87%, respectively (Supplementary file 2).

### Functional Analysis Within the Different Time Point Groups

**Figure 2:**
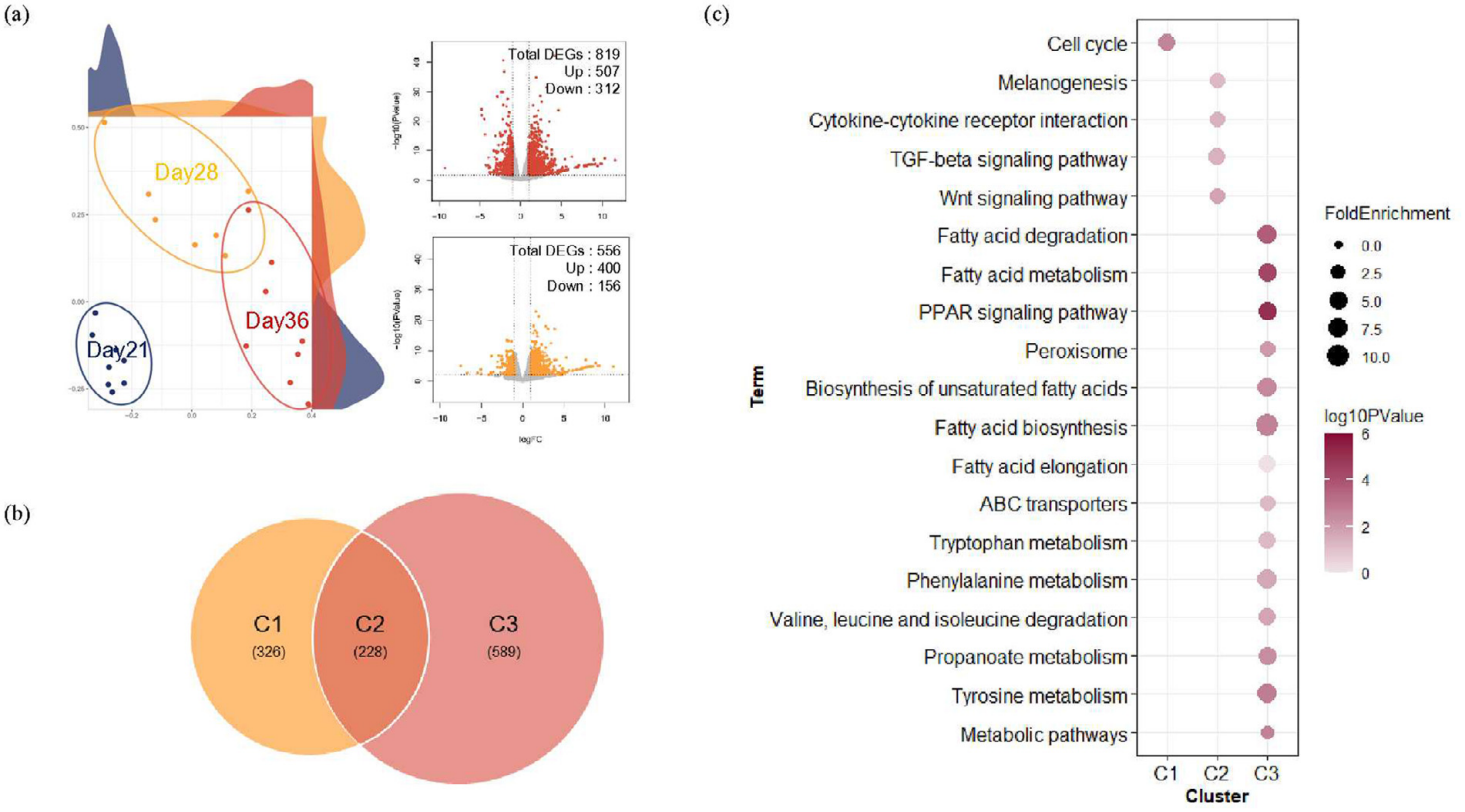
Differentially expressed gene (DEG) profiling and functional analysis at different timepoints. (A) Multidimensional scaling plot reveals differences in transcript expression patterns between d 21, d 28, and d 36. The x and y axes of the volcano plots show the log_2_ fold changes (FCs) and −log_10_*P* values, and DEGs (false discovery rate < 0.05 and log_2_ FC ≥ 1; Up, log_2_ FC ≤ −1; Down) are expressed in color. (B) Visualization of overlapped DEGs using a Venn diagram. (C) Dot plot showing the biological meaning of DEGs within each gene cluster based on the DAVID database as determined following enrichment analysis.

Transcriptomes were compared between the different timepoints. The MDS revealed that each group was separately clustered (Figure 2A). The DEGs were identified by comparing the gene expression levels at D28A and D36A relative to those at D21A. For D36A, 507 and 312 DEGs were up- and downregulated, respectively; 400 and 156 DEGs were up- and downregulated for D28A (Figure 2A). Volcano plots revealed that DEGs increased over time. The overlapping DEGs between D28 and D36 were illustrated using Venn diagrams; each group is annotated as C1 (n = 326), C2 (n = 228), or C3 (n = 589) (Figure 2B). Functional enrichment analyses were performed using KEGG to investigate the biological processes associated with the identified DEGs (Figure 2C). The DEGs included in C1 were enriched in “cell cycle,” a term related to apoptosis and growth. The DEGs included in C2 were enriched in “TGF-beta signaling pathway,” “Wnt signaling pathway,” “cytokine–cytokine receptor interaction,” and “melanogenesis,” which are terms related to cell growth and immune function. The DEGs included in C3 were enriched in “fatty acid degradation,” “fatty acid metabolism,” “PPAR signaling pathway,” “metabolic pathway,” “tyrosine metabolism,” and “propanoate metabolism,” which are terms related to fatty acid and amino acid metabolism.

### Gene Modulation Affecting Significant Pathways at Different Timepoints

**Figure 3:**
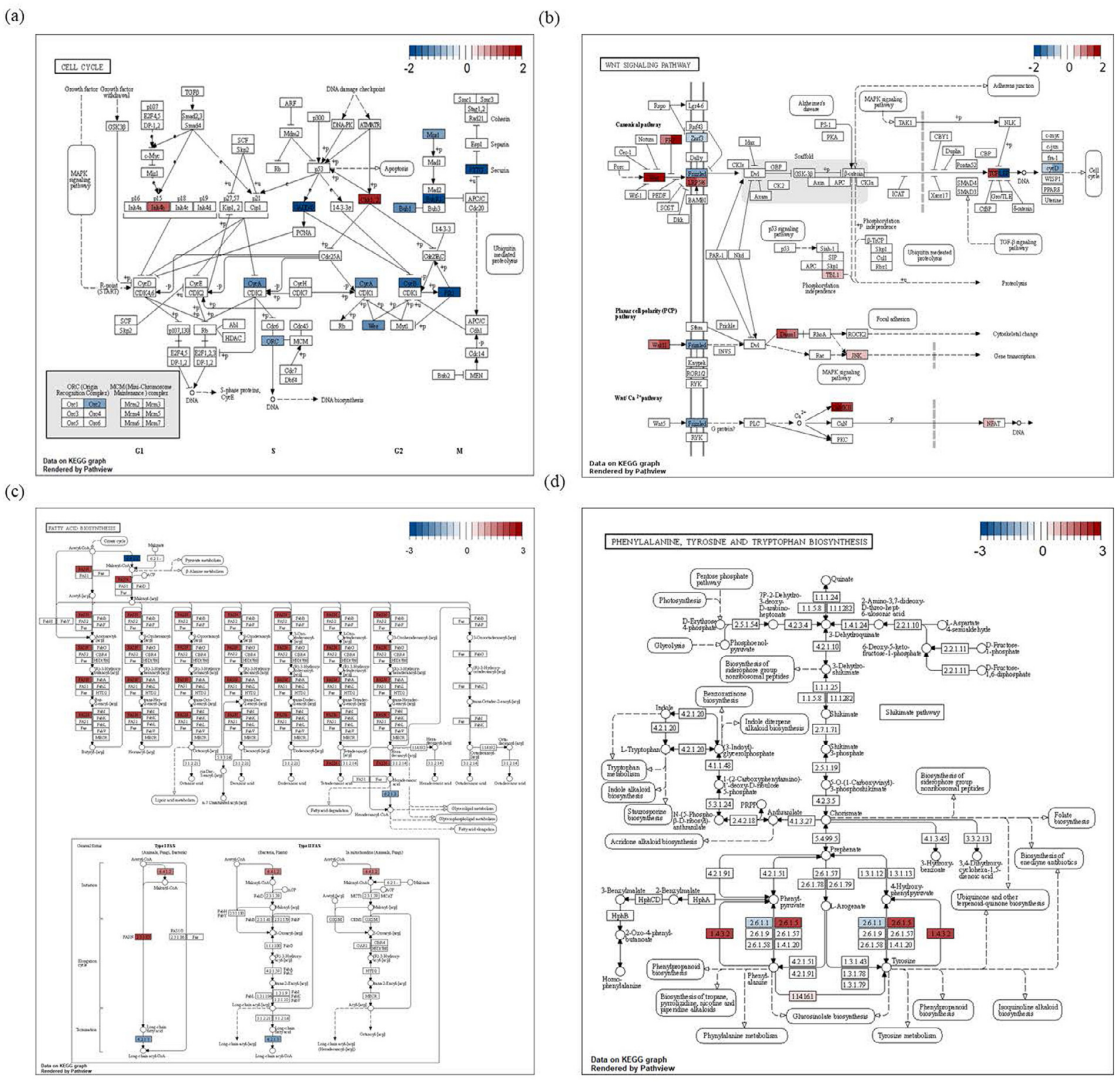
Gene modulation associated with significant Kyoto Encyclopedia of Genes and Genomes (KEGG) pathways at different timepoints. (A) Gene modulation was determined based on gene expression values in the “cell cycle pathway” enriched specifically in C1. (B) Gene modulation in the “Wnt signaling pathway” enriched in C2. (C, D) Gene modulation in the “fatty acid biosynthesis” and “phenylalanine, tyrosine, and tryptophan biosynthesis” pathways enriched in C3.

To identify the gene modulation influencing the functional pathways established based on KEGG, ‘clusterProfiler’ was applied using FC values for the different timepoints. Evaluation of the expression variance associated with the “cell cycle” term for C1 identified that 7 DEGs (*BUB1, TTK, BUB1B, CHEK1, CCNA2, WEE1*, and *ORC2*) related to G2 and M phase were downregulated (Figure 3A). Evaluation of the “Wnt signaling pathway” for C2 identified 7 DEGs (*TCF7, SFRP1, LEF1, WNT11, CAMK2B, DAAM1*, and *WNT2B*), with the expression of *TCF7* and *LEF1* being altered (Figure 3B). Evaluation of “fatty acid biosynthesis” for C3 identified 5 DEGs (*FASN, ACACA, ACACB, ACSL5*, and *ACSBG1*), with *FASN* being upregulated and the remaining 4 DEGs downregulated. In addition, 2 DEGs (*TAT* and *IL4I1*) were upregulated in the “phenylalanine, tyrosine, and tryptophan biosynthesis pathway” for C3 (Figures 3C and 3D).

### Functional Analysis of Different BW Groups at D36

**Figure 4:**
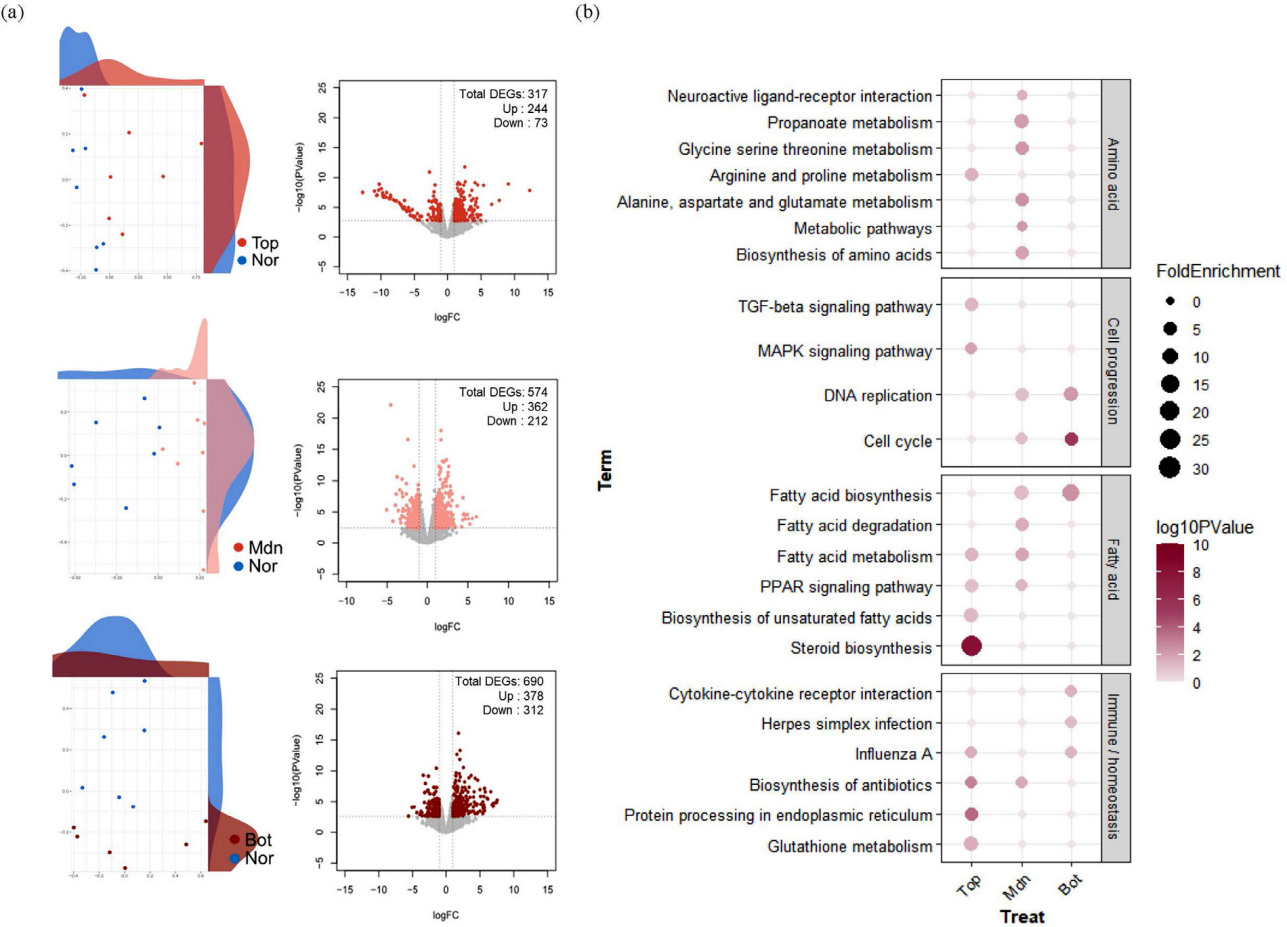
Differentially expressed gene (DEG) profiling and biological functional analysis for the groups with different BW. (A) Multidimensional scaling plot showing differences in transcript expression patterns between D36T (top 10%), D36A (average), and D36B (bottom 10%) compared to D36N. The x and y axes of the volcano plots show the log_2_ fold changes (FCs) and −log_10_*P* values, and DEGs (false discovery rate < 0.05 and log_2_ FC ≥ 1; Up, log_2_ FC ≤ −1; Down) are expressed in color. (B) Dot plot showing the biological meaning of DEGs within each group based on the DAVID database as determined using enrichment analysis.

We obtained liver tissues from the day-36 groups with different BWs and analyzed the associated transcriptomes. The MDS plots show differences in gene expression between TN (D36N) and HS groups (D36T, D36A, and D36B). The DEGs were selected by comparing the gene expression levels at D36T, D36A, and D36B with those at D36N. For D36T, 244 and 73 genes were up- and downregulated, respectively; for D36A, 362 and 212 genes were up- and downregulated, respectively; for D36B, 378 and 312 genes were up- and downregulated, respectively (Figure 4A). Thus, more genes were differentially expressed in both directions in the lower BW group.

Pathway enrichment analyses were performed based on KEGG to investigate the associated biological processes (Figure 4B). All terms were categorized into 4 biological functions: “amino acid metabolism,” “cell progression,” “fatty acid metabolism,” and “immune/homeostasis.” The DEGs in D36T were involved in terms associated with “immunity” and “fatty acid metabolism” (i.e., “steroid biosynthesis,” “fatty acid metabolism,” “PPAR signaling pathway,” “biosynthesis of unsaturated fatty acids,” “glutathione metabolism,” “protein processing in the endoplasmic reticulum,” and “biosynthesis of antibiotics”). The DEGs in D36A were related to “amino acid metabolism” (i.e., “biosynthesis of amino acids,” “metabolic pathway,” “glycine serine,” “threonine metabolism,” “propanate metabolism,” “alanine metabolism aspartate,” and “glutamate metabolism”). The DEGs in D36B were enriched for “cell progression” (i.e., “cell cycle” and “DNA replication”).

### Gene Modulation Affecting Significant Pathways in the Different BW Groups

**Figure 5:**
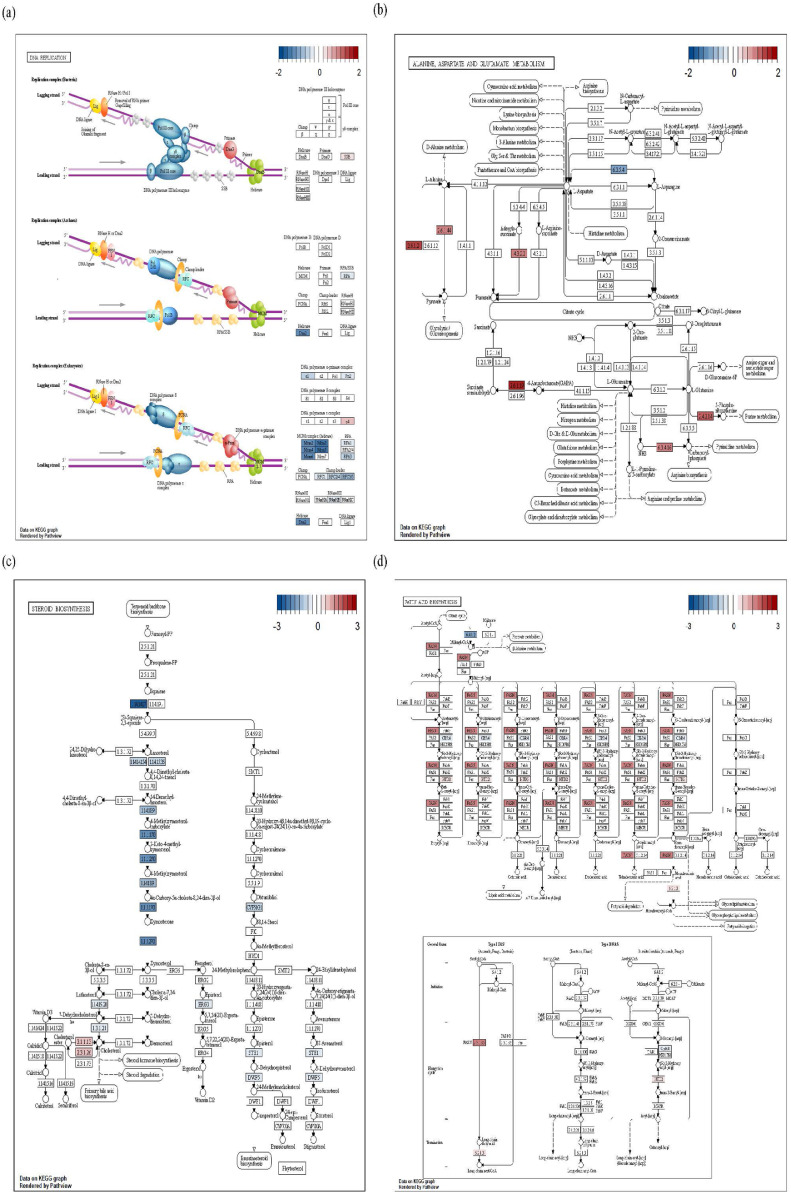
Gene modulation associated with significant Kyoto Encyclopedia of Genes and Genomes (KEGG) pathways in the different BW groups on d 36. (A) Gene modulation was determined based on gene expression values in the “cell replication pathway” enriched specifically in the D36B (bottom 10%) group. (B) Gene modulation in the “alanine, aspartate, and glutamate metabolism” enriched in D36A (average). (C, D) Gene modulation in the “fatty acid biosynthesis and steroid biosynthesis pathway” enriched in D36T (top 10%).

Gene modulations influencing biological function were identified using the KEGG database according to the FC levels for the same-age groups (Figure 5). The terms for “DNA replication” and “cell cycle” were identified as the most significant in D36B. Evaluation of “DNA replication” for D36B identified 5 DEGs (*MCM2, MCM3, MCM4, MCM5*, and *MCM6*) related to the mini-chromosome maintenance complex (helicase) that were downregulated (Figure 5A). For D36A, “alanine, aspartate and glutamate metabolism” was the most significant term, with 6 associated DEGs (*ABAT, ADSL, AGXT2, CPS1, GPT2*, and *PPAT*) that were upregulated (Figure 5B). For D36T, significant terms were mainly related to “fatty acid metabolism,” with “steroid biosynthesis” as the most significant pathway. Specifically, 6 DEGs (*MSMO1, SQLE, HSD17B7, FDFT1, DHCR24*, and *NSDHL*) related to zymosterol, a precursor and storage state of cholesterol, were downregulated, and 2 DEGs (*LIPA* and *SOAT1*) involved in cholesterol production were upregulated (Figure 5C). In the “fatty acid biosynthesis” pathway, only upregulation of *FASN*, related to fatty acid production, was observed (Figure 5D).

### Functional Analysis of Genes Common to the Three Groups With Different BW

**Figure 6:**
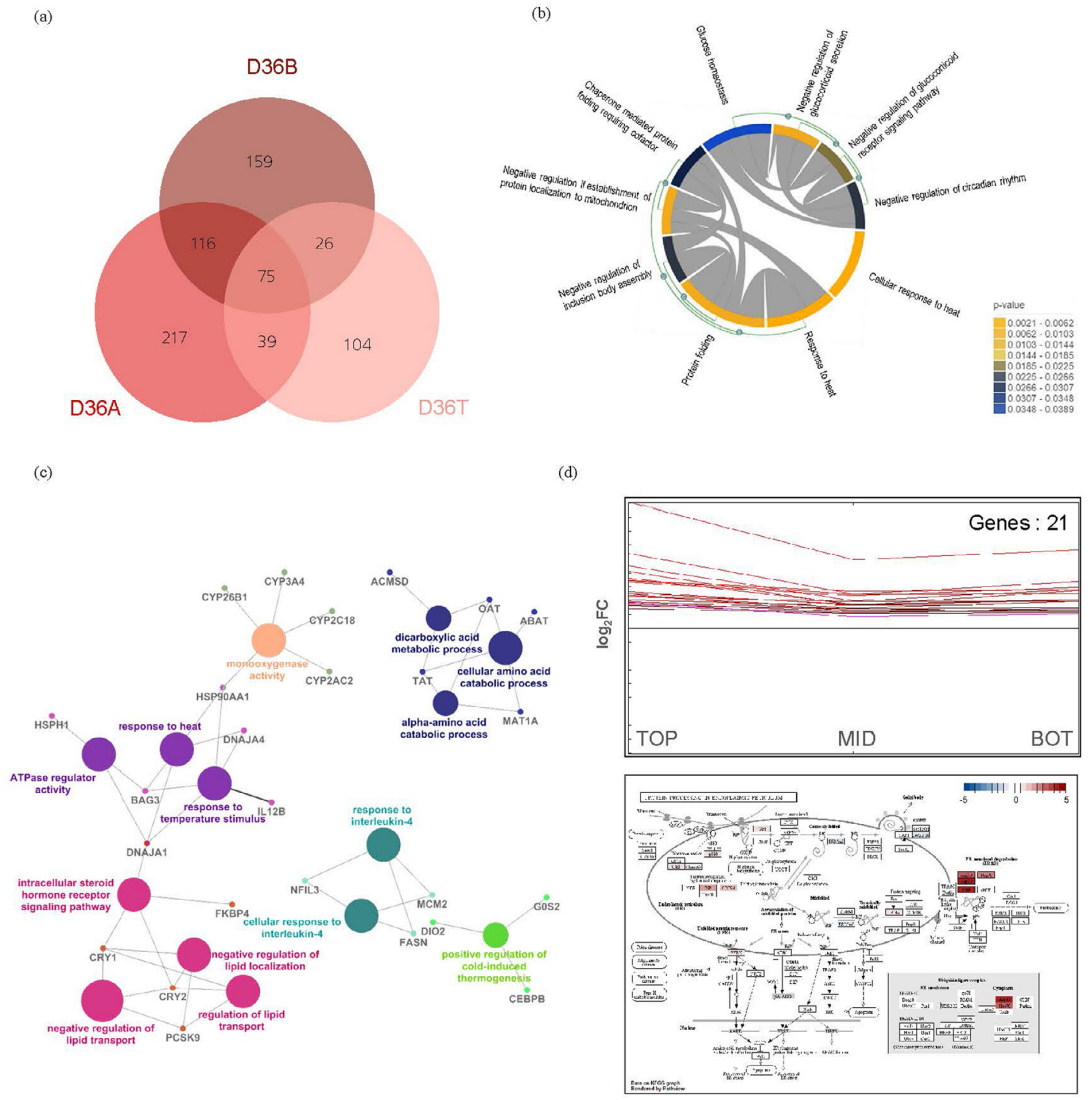
Functional analysis of common genes among the three groups with different BW (D36T [top 10%], D36A [average], and D36B [bottom 10%]). (A) Visualization of overlapping differentially expressed genes using Venn diagrams. (B) MonaGO was used with the Gene Ontology database to visualize genes that interacted with each pathway. (C) The ClueGO (v. 2.5.5, *P*-value ≤ 0.05, Kappa score = 0.4) plugin was used with the Kyoto Encyclopedia of Genes and Genomes (KEGG) database to visualize genes related to each pathway. (D) Genes upregulated in the D36T group and their biological functions based on the gene expression pattern calculated using Multiple Experiment Viewer.

Overlapping DEGs among the same-age groups are shown in a Venn diagram (Figure 6A). There were 104, 217, and 159 specific DEGs in the D36T, D36A, and D36B groups, respectively; the biological functions are listed in Supplementary file 3. A total of 75 common DEGs were identified (Figure 6A). To establish the biological functions of the 75 DEGs, we performed enrichment analysis based on the GO term database and visualized the results (Figures 6B and 6C).

The terms from MonaGO “response to heat,” “protein folding,” and “chaperone-mediated protein folding requiring cofactor” formed a network related to genes responding to HS. Moreover, “negative regulation of glucocorticoid secretion” and “glucocorticoid receptor signaling” were related to glucocorticoid secretion. The constructed network among terms “response to heat,” “response to temperature stimulus,” and “ATPase regulator activity” was related to heat response, whereas that for “negative regulation of lipid transport” and “regulation of lipid transport” was related to fat metabolism. Furthermore, “alpha-amino acid catabolic process” and “cellular amino acid catabolic process” were related to amino acid metabolism. Among the 75 genes, 21 were upregulated to a greater degree in D36T. Enrichment analysis based on the KEGG database identified the term “protein processing in the endoplasmic reticulum pathway” as significant and revealed that the heat shock protein (**HSP**) gene in this pathway was upregulated (Figure 6D).

### QPCR Validation of Gene Expression and the PPI Network

**Figure 7:**
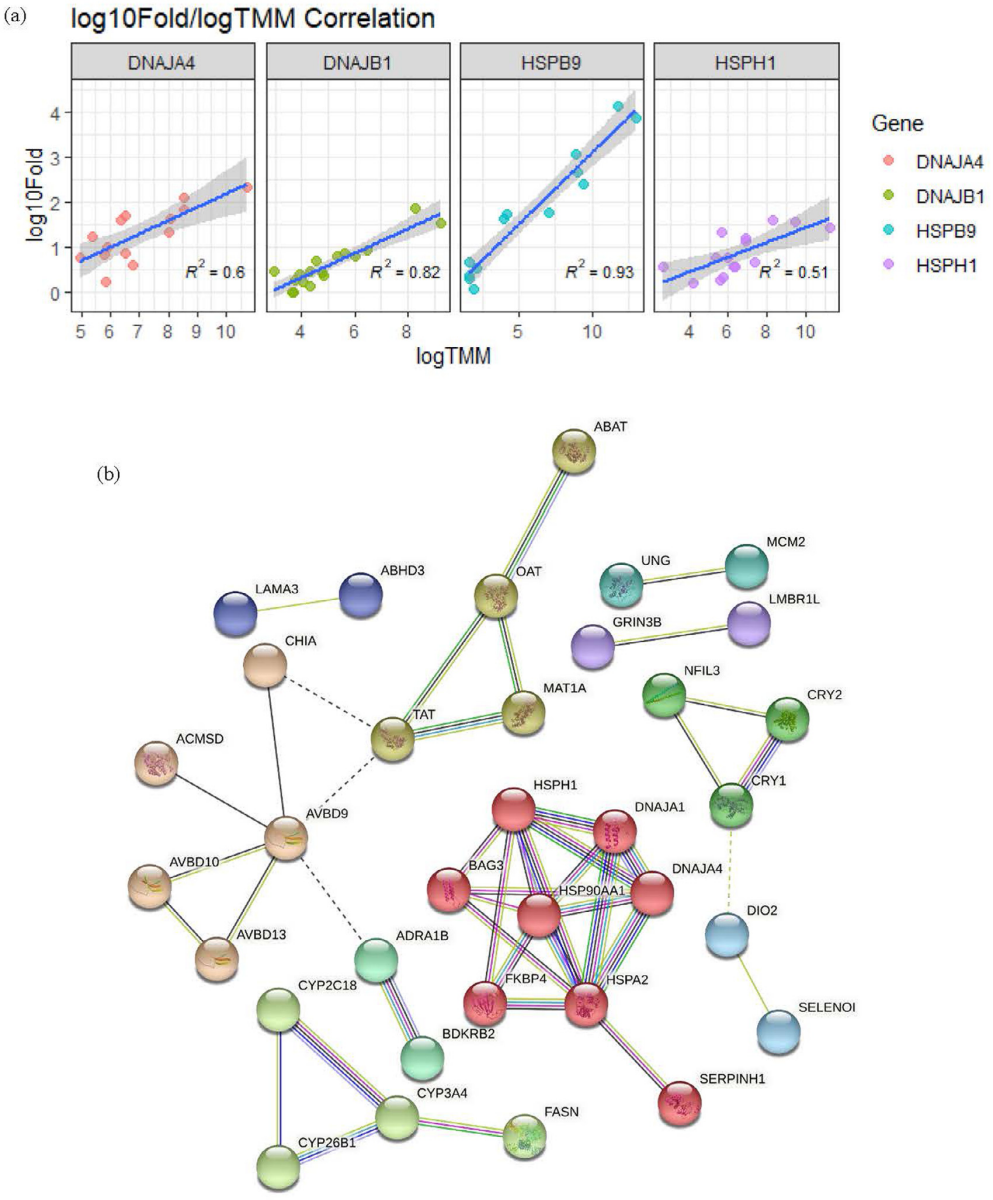
qPCR validation of gene expression and the protein–protein interaction (PPI) network. (A) Regression plot displaying the direct correlations between the logTMM and log_10_ fold change values of heat stress proteins. (B) The PPI network showing interactions between proteins based on gene expression. TMM, trimmed mean of M-value.

To technically validate genes with specifically higher expression in D36T than in the other groups, we performed qPCR analysis and compared the resultant gene expression to that determined using RNA sequencing. We selected 4 genes (*DNAJA4, DNAJB1, HSPB9*, and *HSPH1*) included in the significant biological pathway “protein processing in the endoplasmic reticulum.” To quantitatively evaluate the expression, we calculated the Pearson's correlation coefficients using the logarithmic ratio of change between the 2 experiments. We observed significant correlations: *DNAJA4* (R^2^ = 0.6), *DNAJB1* (R^2^ = 0.82), *HSPB9* (R^2^ = 0.93), and *HSPH1* (R^2^ = 0.51; Figure 7A). A PPI network was then constructed using the 21 genes that were specifically upregulated in the D36T group. The protein groups showing the highest PPI score based on expression levels were HSPH1, DNAJA1, DNAJA4, HSP90AA1, FKBP4, BDKRB2, HSPA2, and SERPINH1 (Figure 7B).

## DISCUSSION

### Growth Performance and Common Biological Pathways

Broiler chickens have been found to modify their physiological state to remain viable under HS conditions. Organisms become acclimatized when exposed to continuous high-temperature environments, with genetic differences being strongly correlated with susceptibility to and tolerance of HS (Nadel et al., 1974; Baum et al., 1976; Lim et al., 2020). Despite this knowledge, a comprehensive understanding of the mechanisms underlying HS response and acclimation at the molecular level is lacking.

The current study compared gene expression levels in 2 dimensional comparisons (differences in timepoint and BW) under cyclic HS. A significant difference in final BW was observed under HS conditions on d 36, indicating that our experimental conditions were sufficient to induce HS in broilers. Moreover, differences in BW were observed among broilers raised under the same cyclic HS conditions. These results are likely associated with susceptibility or tolerance, which are closely related to temperature acclimation. Based on MDS analysis, we identified a well-defined trajectory of transcriptomes related to HS in each group. The significant biological terms common between the 2 comparisons included “cell growth,” “fatty acid metabolism,” “amino acid metabolism,” and “immune/homeostasis.”

Broiler chickens acclimatize to HS conditions during growth (Renaudeau et al., 2012). Thus, the metabolic changes at different time points and significantly enriched biological terms indicate the mechanisms underlying acclimation. Moreover, differences in growth performance under the same high-temperature conditions indicate the adaptation mechanism (Pamok et al., 2009). Notably, a decrease associated with the “cell cycle” and increased “fatty acid or amino acid metabolism” were commonly identified in higher growth and weight broiler groups. It could, therefore, be assumed that both groups were acclimatized to HS conditions.

### Cell Cycle Arrest As an Indicator of Non-acclimation Under HS

Mammalian cells temporarily arrest primarily at 2 cell cycle checkpoints, the G1/S and G2/M transitions, when exposed to stressed conditions to repair damaged DNA (Kühl et al., 2000). In both the C1 and D36B groups, the “cell cycle” pathway biological term was enriched. In C1, gene modulation analysis revealed that genes related to the G2/M phase were downregulated. However, in D36B, genes associated with all cell cycle phases were downregulated. The term "DNA replication" from D36B was most significant, and *MCM2, MCM3, MCM4, MCM5*, and *MCM6* were downregulated (Maiorano et al., 2006). The protein encoded by this gene is a highly conserved mini-chromosome maintenance protein involved in the initiation of eukaryotic genome replication. In C1, cell growth is expected owing to the age differences between samples; however, as all broilers in D36B were of the same age, the results revealed the existence of a limitation not only in cell growth but also in DNA replication in the G1/S phase. This observation can be attributed to a mechanism that restricts DNA replication (indicating cell growth) when the DNA is damaged (Sun et al., 2015; Velichko et al., 2015). Therefore, these results indicate that HS causes a physiological imbalance in cells and is related to cell cycle arrest or cell apoptosis, which results in cell death distinct from that resulting from DNA replication in an unstable state in the broiler liver.

Furthermore, in C2, the *TCF7* and *LEF1* genes exhibited modulation in the “Wnt signaling pathway,” which regulates the cell cycle. These genes were downregulated over time from an initial upregulated state, which may underlie the response mechanism to HS. Notably, the term “Wnt signaling” does not imply a single-purpose signal transduction system. Rather, it refers to various signals triggered by Wnt ligand-receptor interactions that direct cell behavior in multiple ways: cell polarity, movement, proliferation, differentiation, survival, and self-renewal. Expression of TCF and LEF in cells can block Wnt signaling and force cell cycle arrest in the G1 phase (Wu et al., 2012).

In the C1 and D36B groups, the terms related to energy metabolism for normalizing the status of broilers exposed to HS conditions could not be identified; only the cell cycle arrest term could be confirmed (Cadigan and Waterman 2012; Ye et al., 2019). These findings imply that the C1 and D36B groups exhibited difficulty in acclimating to HS.

### Energy Metabolism Activity Through Amino Acid Catabolism

There are abundant nonessential amino acids in the liver, such as alanine, aspartate, glutamate, glycine, and serine, and essential amino acids, such as histidine and threonine. These amino acids are involved in various cellular metabolism processes, the synthesis of lipids and nucleotides, and detoxification reactions (Lee and Kim, 2019). The list of significant terms related to amino acid metabolism was highly enriched in C3 and D36A. The DEGs of group C3 were associated with the term “phenylalanine tyrosine and tryptophan biosynthesis.” *TAT* and *IL4L1* are respectively involved in converting phenylalanine and tyrosine into the metabolite pyruvate acid before being used as energy in the TCA cycle (Jantzen et al., 1987; Rapoport et al., 2008). *TAT* is a mitochondrial protein, tyrosine aminotransferase, which presents in the liver and catalyzes the conversion of L-tyrosine into p-hydroxy phenylpyruvate. In turn, the D36A group was linked with terms “alanine aspartate and glutamate metabolism” and “glycine serine and threonine metabolism,” with genes (*GPT2, ADSL, AGXT2*, and *ABAT*) in these pathways being upregulated. *GPT2* and *AGXT2* catalyzed the reversible transamination between alanine and 2-oxoglutarate to generate pyruvate and glutamate (Dyson et al., 2015). Moreover, *ADSL* and *ABAT* converted amino acids into fumarate and succinate for use in the TCA cycle (Medina-Kauwe et al., 1999; Vozarova et al., 2002; Spiegel et al., 2006; Caplin et al., 2012). Identification of the modulated DEGs associated with these enriched terms highlighted the biological mechanism of energy use by breaking down amino acids. According to previous studies, HS results in decreased feed intake and elevated respiratory rate in broilers, subsequently inducing glycogen depletion and a negative energy balance (Emami et al., 2021). As the liver requires a high rate of energy metabolism under HS conditions, this result suggests that the liver undergoes increased catabolism to reach homeostasis for energy demands (Lan et al., 2016).

### Fatty Acid Degradation is Utilized in Heat Adaptation Mechanisms

Fatty acid metabolism is an essential mechanism closely related to energy homeostasis under HS conditions. Numerous terms related to fatty acid metabolism were associated with the DEGs of C3, and significant terms also appeared in different BW groups on d 36. Compared to that in the C3 group, the expression level of *FASN* was decreased in D36T, whereas *ASVR1C* was upregulated. Upregulation of *FASN*, a representative fatty acid synthesis gene, results in increased fatty acid synthesis. Conversely, increased *ASVR1C* can induce the breakdown of fatty acids providing energy to the liver. The most significantly enriched term was “steroid biosynthesis” (Figure 4B). The prominent regulatory trends within this term reflected downregulation of zymosterol-related genes (*MSMO1, SQLE, HSD17B7, FDFT1, DHCR24*, and *NSDHL*) and upregulation of cholesterol-producing genes (*LIPA* and *SOAT1*).

The liver generally attempts to absorb or break down fatty acids to generate energy upon continuous exposure to HS (Shim et al., 2006). However, hormone regulators, such as glucocorticoids, interfere with this mechanism (Hara et al., 2014; Rahimi et al., 2020). As the majority of fat stored in avian adipose tissue is derived from very low density lipoprotein (**VLDL**) supplied by the liver, abnormal fat deposition in heat-stressed broiler chickens likely results from effects on hepatic lipid synthesis (Peterson et al., 1990). In D36A, terms related to catabolism of amino acids were enriched. In comparison, in D36T, terms associated with fatty acid degradation were enriched. This may be linked to the formation of VLDL, a type of excretion. During the initial adaptation to HS, increased transport of VLDL to adipose tissue appears to match the increased rate of lipid synthesis. However, sustained HS may overwhelm the ability of the liver to export sufficient lipids, as reflected in significant increases in liver weight and triglyceride content in broilers (Wasti et al., 2020). Moreover, we confirmed that genes related to cholesterol production were upregulated in D36T, whereas those involved in zymosterol storage were downregulated. This result implies that fatty acid degradation and release in D36T were relatively enhanced compared to this functionality in other HS groups. Therefore, it can be hypothesized that these regulatory mechanisms are involved in acclimation through the highest caloric energy-producing hepatic process by increasing fatty acid catabolism. The acclimation mechanism was demonstrated in the high-BW group.

### HSP-Induced Homeostasis and Heat Acclimation

Venn diagrams illustrated the similarities and differences in gene expression among different growth performance groups at D36. Notably, the results did not substantively differ from those obtained by analyzing biological terms for each treatment group. In particular, the 75 genes common between the groups were associated with the mechanism of response to heat, glucocorticoid secretion, and energy metabolism.

Acclimation to heat refers to an organism's ability to perform in elevated environmental temperatures and survive in the presence of elevated but nonlethal core temperatures (Fruth and Gisolfi 1983). Heat acclimation minimizes the enhancement of heat transfer to the skin or heat-dissipating capacity and allows the organism to tolerate a higher core temperature (Xie et al., 2014). This involves a reduction in the sweating threshold (the sweating output at a given core temperature), a reduced point for cutaneous vasodilation, and greater skin blood flow at a given core temperature to increase heat dissipation through systemic alterations (Tutar and Tutar 2010).

Twenty-one genes were specifically upregulated in the D36T group. Functional analysis of D36T revealed the same HSP-related terms associated with the functions of these 21 genes. Recent studies have reported a relationship between HSPs and heat acclimation (Feder and Hofmann, 1999). The HSPs play vital roles in cell tolerance to HS through mechanisms including denatured protein management, cytoskeletal stabilization, protein translocation across membranes, and receptor regulation (Siddiqui et al., 2020). Overexpression of various HSPs confers tolerance in the absence of conditioning stress, whereas inhibition of HSP accumulation by blocking antibodies impairs stress tolerance, which supports the hypothesis that HSPs themselves confer stress tolerance (Collier et al., 2019). Although the mechanism by which HSPs confer stress tolerance is not completely understood, it may be related to the important role of HSPs in the establishment of homeostasis.

Glutathione metabolism, a precursor of glutathione peroxidase, an important oxidizing agent, also showed significance in D36T. This observation could explain why the expression of oxidase, a major indicator of homeostasis under HS, was expressed (Payne and Southern, 2005), which in turn suggests that HSPs improved the heat acclimation mechanism in D36T broilers. Notably, in addition to our general results, supported by the validation of RNA sequencing data through gene expression analysis using qPCR, when protein interactions were confirmed using PPI network constraints, HSPs showed the highest interaction score, further confirming their relevance to the physiological adaptations in the acclimated broilers.

## CONCLUSIONS

The mechanism underlying acclimation to HS conditions in broilers was investigated by comparing differences in two-dimensional transcriptome profiles in liver tissues. Our study identified “fatty acid degradation” and “HSP expression” as being upregulated in the higher growth and weight groups, whereas “cell cycle arrest” and “amino acid metabolism” were downregulated. The higher BW group that indicated acclimation supplied fat and energy from the liver to tissues through the breakdown of fatty acids. Furthermore, homeostasis was maintained via HSPs and antioxidant enzymes. The significant candidate genes and regulatory mechanisms associated with the response to heat might serve as a foundation for improving the ability of chickens to acclimatize under HS.

## Appendix Supplementary materials

**Additional file 1:** Table S1. RNA quality scores. Image, application 1

**Additional file 2:** Table S2. Overview of data processing. Image, application 2

Image, application 3
