# Supplementary material for: Hepatic transcriptome profiling according to growth rate reveals acclimation in metabolic regulatory mechanisms to cyclic heat stress in broiler chickens
Source: Poult Sci. 2022 Sep 20;101(12):102167. doi: 10.1016/j.psj.2022.102167 (PMC9579409; doi:10.1016/j.psj.2022.102167)
Supplement: Supplementary file 3 [file mmc3.docx]

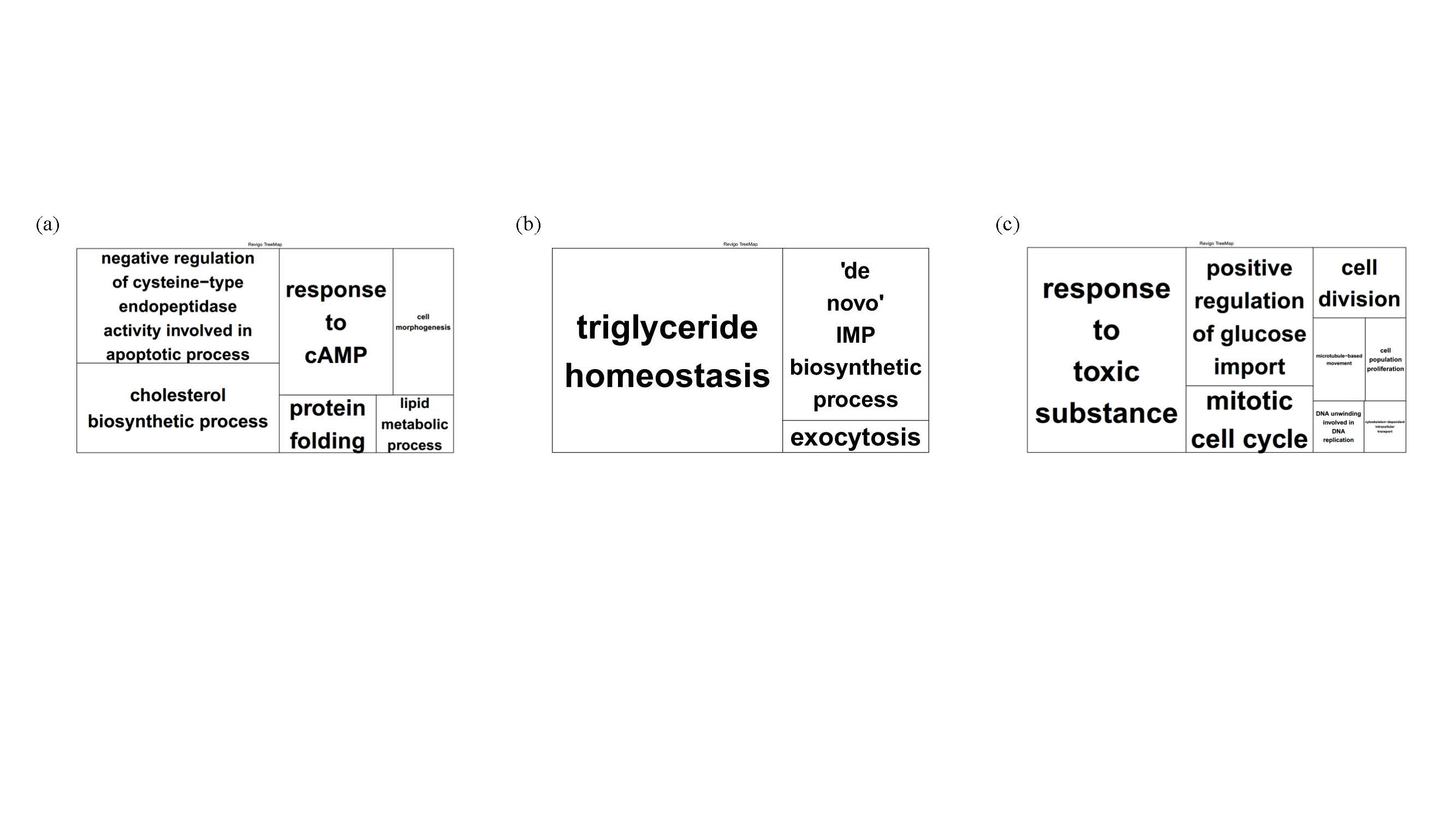


**Additional file 3:** Fig. S1 Gene Ontology treemaps created based on *P*-values for biological process terms. Functional analysis of specific differentially expressed genes for each group was performed: (a) D36T (top 10%), (b) D36A (average), and (c) D36B (bottom 10%)
